# The role of arthroscopy in intra‐articular adipose tissue‐derived mesenchymal stem cells for the treatment of early‐stage knee osteoarthritis: A bicentric retrospective comparative study

**DOI:** 10.1002/jeo2.70368

**Published:** 2025-07-21

**Authors:** Simone Giusti, Ezio Adriani, Kristian Samuelsson, Alice Laudisio, Alexandra Horvath, Biagio Zampogna, Rocco Papalia

**Affiliations:** ^1^ Unita Operativa Complessa di Traumatologia dello Sport e Chirurgia Articolare Fondazione Policlinico Universitario Agostino Gemelli IRCCS Rome Italy; ^2^ Operative Research Unit of Orthopaedic and Trauma Surgery Fondazione Policlinico Universitario Campus Bio‐Medico Rome Italy; ^3^ Research Unit of Orthopaedic and Trauma Surgery, Department of Medicine and Surgery Università Campus Bio‐Medico di Roma Rome Italy; ^4^ Università Cattolica del Sacro Cuore Rome Italy; ^5^ Department of Orthopaedics Sahlgrenska University Hospital Mölndal Sweden; ^6^ Department of Orthopaedics, Institute of Clinical Sciences, Sahlgrenska Academy University of Gothenburg Gothenburg Sweden; ^7^ Department of Biomedical, Dental, Morphological and Functional Images University of Messina, A.O.U. Policlinico “G.Martino” Messina Italy

**Keywords:** arthroscopy, chondral lesions, knee, knee osteoarthritis, mesenchymal stem cells

## Abstract

**Purpose:**

To determine the difference in clinical scores and re‐intervention rates in patients receiving intra‐articular adipose‐derived mesenchymal stromal cells (AD‐MSCs) as a stand‐alone treatment for knee osteoarthritis (OA) compared to patients receiving the same treatment following arthroscopic debridement and lavage.

**Methods:**

Internal records at two orthopaedic centres were reviewed, and all consecutive patients with Kellgren–Lawrence II–III knee OA who had received intra‐articular AD‐MSC during 2017–2018 were included. The patients were stratified into two cohorts depending on whether they also received debridement arthroscopy. Patients were evaluated with the Western Ontario and McMaster Universities Osteoarthritis Index (WOMAC) and visual analogue scale (VAS) scores as well as re‐intervention rates with a last available follow‐up of 5 years after the intra‐articular AD‐MSC injection.

**Results:**

A total of 135 patients were enroled, 66 (49%) patients were male and 69 (51%) were female. The mean age at the time of intervention was 66 (range: 43–81) years. About half of the cohort (*n* = 68) received a diagnostic and therapeutic arthroscopy (intervention) procedure in the same setting, whereas the other half (*n* = 67) received intra‐articular AD‐MSCs without an arthroscopic procedure (control). Totally, 94% reported improved VAS scores post‐operatively. Seventy‐two patients (53%) had good symptomatic control at 5 years after the intra‐articular AD‐MSCs injection (VAS score range 0–3). Within this group, 57% of the patients had also received arthroscopy as part of their treatment, whereas the remaining patients had only received the intra‐articular injection of AD‐MSCs. Overall, WOMAC (46 arthroscopy + AD‐MSC, 58 AD‐MSC, *p* < 0.0001) and functionality (34 arthroscopy + AD‐MSC, 43 AD‐MSC, *p* < 0.0001) scores were superior in the cohort who also received debridement arthroscopy at the 5‐year follow‐up. Conservative re‐intervention rates, such as hyaluronic acid injections, were comparable amongst the cohorts.

**Conclusion:**

Overall, debridement arthroscopy with AD‐MSC is favoured over stand‐alone MSC in Kellgren–Lawrence I–III knee OA.

**Level of Evidence:**

Level III, retrospective comparative study.

**Abbreviations**
AD‐MSC: adipose‐derived mesenchymal stromal cell
BMI: body mass index
OA: osteoarthritis
PRP: platelet‐rich plasma
RCT: randomised controlled trial
VAS: visual analogue scale
WOMAC: Western Ontario and McMaster Universities Osteoarthritis Index

## INTRODUCTION

Knee osteoarthritis (OA) is a chronic, degenerative condition that primarily affects the elderly population. As of 2020, knee OA was regarded as the fourth worldwide leading cause of disability in the ageing population, affecting 15% of people over 65 years of age [3]. Knee OA is characterized by the gradual destruction of articular cartilage (specifically the extracellular matrix), which eventually involves femoral, tibial and in some cases patellar articular surfaces. The results of this condition are primarily chronic pain and loss of functionality. Articular cartilage cannot regenerate after being irreparably damaged, as it is not sufficiently vascularized. This explains why, to date, the only definitive treatment remains surgical replacement of the knee joint and why OA remains refractory to most non‐surgical treatments [4]. Since the early 2000s, the research community has supported the use of orthobiologics such as mesenchymal stromal cells (MSCs) and plasma‐derived growth factors with the aim of controlling intracellular processes by modifying or activating cell function. This is believed to contribute to more efficient and rapid tissue repair. More specifically, MSCs, due to their capability of self‐regeneration and differentiation to replace damaged and dying cells through paracrine or signalling function, were found to hold extraordinary potential in a non‐surgical treatment for OA [16]. In fact, previous findings demonstrate how intra‐articular MSCs have the potential to induce hyaline‐like cartilage regeneration [19]. The most abundant sources of MSCs derive from autologous adipose tissue, bone marrow, or umbilical cord tissue. Multiple studies have determined the effectiveness of intra‐articular MSCs injections for treating early‐stage OA, as defined by Luyten et al. [13] over the years, providing encouraging data in the short term [20]. However, MSCs have yet to become a definitive solution for this subset of patients, as long‐term results are absent following MSC injections. The main goal of the current study was to assess whether differences exist in clinical scores and re‐intervention rates in patients receiving intra‐articular adipose‐derived (AD)‐MSCs as a stand‐alone (control) treatment for knee OA compared to patients receiving the same treatment following arthroscopic debridement and lavage (intervention). It was hypothesized that those who received arthroscopy would obtain better outcomes and a lower re‐intervention rate than patients without arthroscopy.

## MATERIALS AND METHODS

### Participants and study site

This is a retrospective study performed at two of Italy's largest orthopaedic centres: Fondazione Policlinico Universitario Agostino Gemelli (Rome) and Fondazione Policlinico Universitario Campus Bio‐Medico (Rome). A retrospective chart review of the medical records at both hospitals was performed, including patient consultations, operative notes, and patient‐specific databases. This entailed all patients who had received intra‐articular AD‐MSC, both with arthroscopy (intervention group) and without (control), from 1 January 2017 to 31 December 2018, allowing for at least 5 years of follow‐up. Consecutive patients who had received intra‐articular AD‐MSCs (adipose‐derived) injections over the 2017–2018 period were eligible for inclusion. All patients had radiographic evidence of knee OA. Pre‐operative imaging was used to subdivide patients according to the severity of their knee OA, ranging between Grade II (mild OA) and Grade III (moderate OA) of the Kellgren–Lawrence radiographic scale (Table 1) [8]. Patients with axial deviations in varus or valgus greater than >5° were excluded from the study.

**Table 1:** Patients subdivided by the Kellgren–Lawrence osteoarthritis severity scale.

	Grading			
	I	II	III	IV
Patients (*n*, %)	0	98 (72.5)	37 (27.4)	0

### Study outcomes

Patient data was extrapolated from medical charts, including age, sex, body mass index (BMI), pre‐intervention visual analogue scale (VAS), and comorbidities including ischaemic cardiac pathology, diabetes mellitus, hypertension, peripheral vascular disease, thyroid pathology and hypercholesterolaemia. Patients who had received prior treatment for their OA, including anti‐inflammatory medication, platelet‐rich plasma (PRP) injections, and hyaluronic acid injections, were also identified. All the patients who had not yet received further treatment, including non‐operative management (hyaluronic acid, PRP) or joint replacement surgery for their knee OA, were analyzed with Western Ontario and McMaster Universities Osteoarthritis Index (WOMAC) and VAS scores at the last follow‐up consultation note at 5 years from their intra‐articular AD‐MSCs injection.

### Surgical procedure

The patients who received diagnostic and therapeutic arthroscopy as a first step of the procedure (intervention group) received chondroabrasion of all articular surfaces, loose body removal and articular lavage. No ligamentous reconstruction or meniscal repair/meniscectomy was performed. Simultaneously, a second operator performed two small (1 cm) incisions in the abdomen, in the right and left iliac fossae, and injected Klein's solution (a mixture of Lidocaine 50 mL 1%, Epinephrine 0.5 mL of 1:100,000 and 10 mL of 8.5% Sodium Bicarbonate) into the subcutaneous fat. Klein's solution favours the breakdown of adipose cells [9], decreases bleeding by causing vasoconstriction, and reduces post‐operative pain. Microcannula liposuction was then performed (13G blunt‐suction). The harvested adipose tissue was fragmented and washed in specific closed‐circuit devices, producing an MSC‐rich substance that was injected under direct visualisation into the knee‐dried joint after the arthroscopic debridement phase. Two experienced orthopaedic surgeons performed all injections and arthroscopies. Multiple systems for preparing adipose tissue are available, the most widely used techniques being micro‐fragmentation, filtration, and slow centrifugation, as described by Papalia et al. [15] in their pilot study. The two systems used to prepare the MSC‐rich substance in our study were Lipocell^©^ and Lipogems^©^. Lipocell^©^ technique relies on a semipermeable membrane and continuous irrigation to separate adipocytes and stem cells from waste elements. In contrast, the Lipogems^©^ technique obtains a homogenous filtrate by progressive size reduction of adipose tissue by forced passing of the substrate in multiple size‐reducing nets within a closed chamber. Both these systems extrapolate MSC‐rich concentrates from micro‐fragmented adipose tissue, which has already been proven effective in multiple studies [2]. The patients who did not receive arthroscopy (control group) as a first step of the procedure underwent an identical second phase of AD‐MSCs harvesting and preparation and were injected with the standard anterolateral technique. All patients underwent a non‐weight‐bearing period of 14 days post‐operatively (similar protocols have been used for patients undergoing AD‐MSC injections) [5]. All the patients in the intervention group were hospitalized for one day and were sent home the day after the procedure. In contrast, all the patients who only received intra‐articular MSCs were sent home the same day.

### Statistical analysis

Statistical analyses were performed using IBM SPSS Statistics for Windows, Version 28.0 (IBM Corp.). Data of continuous variables were presented as mean values ± standard deviation (SD). Median values with inter‐quartile ranges were provided for non‐normally distributed variables. Analysis of variance (ANOVA) for normally distributed variables was performed to compare outcomes between groups; otherwise, the non‐parametric Mann–Whitney *U* test was adopted. The two‐tailed Fisher's exact test was used for dichotomous variables. Differences were considered significant at the *p* < 0.05 level.

## RESULTS

### Baseline characteristics

In total, 145 patients receiving AD‐MSC treatment were eligible for the study. Ten patients were lost to follow‐up, resulting in 135 patients being included in the final analyses. Of these, 66 (49%) patients were male and 69 (51%) were female. Approximately half of the patient cohort (*n* = 68, 50.4%) received a diagnostic and therapeutic arthroscopy (intervention) procedure in the same setting, whereas the other half (*n* = 67, 49.6%) received intra‐articular MSCs without an arthroscopic procedure (control). The mean age at the time of intervention was 66 (±8) years, with the youngest patient aged 43 and the oldest 81 years, respectively. The average BMI was 27.7 kg/m^2^, with the lowest recorded BMI being 22.4 and the highest at 41.6 kg/m^2^, respectively. None of the enroled patients had previously undergone intra‐articular AD‐MSCs injections, but all had received previous treatment: 84% (*n* = 113) of the patients had received intra‐articular hyaluronic‐acid injections and 38% (*n* = 51) PRP injections. All patients had symptomatic OA at the time of the intervention, with a mean VAS score of 5 (range 3–7). The average duration for the operative arthroscopy was 16 min (range: 7–32 min). All patients received chondroabrasion of all articular surfaces and articular lavage. Twenty‐three patients (34%) also received loose body removal.

Out of 135 patients, 94% (*n* = 127) reported improvements in VAS scores (average VAS improvement 2.1), whereas 6% (*n* = 8) of the patients did not report any improvement in VAS scores from the received treatment. Of the eight patients who did not benefit from the treatment, five were female, and three were male. Only two of these received AD‐MSCs in addition to arthroscopy. After 12 months, only 1 patient had received surgical treatment, specifically a medial unicondylar knee replacement. This patient had also received arthroscopy as part of the treatment and fell into the ‘Moderate’ Kellgren–Lawrence category. Of the remaining 134 patients at 12 months, 8.1% (*n* = 11) had received additional non‐surgical treatment for recurrent knee pain; specifically, hyaluronic acid injections (these were offered at a different institution). Out of these 11 patients, 6 had not received arthroscopy. Of the total study population, 123 (91.1%) had not received surgical treatment for their knee OA 1 year after the intervention. When searching the internal databases in July 2023, all patients had at least 5 years of follow‐up from the intervention. Currently, 13 (9.6%) patients (5 received arthroscopy, whereas 8 only underwent AD‐MSCs injections) out of the 135 initial cohort have undergone unicondylar or total knee replacement surgery. Of these 13 patients, 4 underwent unicondylar knee replacement, whereas 9 received total knee replacement surgery. The average time from MSCs' intra‐articular injection to surgery in the patients who ended up receiving a knee replacement was 38 months (10–57 months), and 12 out of 13 were satisfied with post‐operative results. At 5 years, 122 (90.4%) of the initial patient cohort had not received surgical treatment for knee OA. We subdivided these patients into groups, representing why they had not received unicondylar or total knee replacement surgery (Table 2).

**Table 2:** Reasons behind not receiving knee replacement surgery at 5 years from intervention.

	Not symptomatic	Unable to undergo surgery for unrelated health issues	Are due to undergo surgery (waiting list)	Are reluctant to undergo surgery (fear of intervention)
*N* (%)	72 (59)	19 (16)	12 (9)	19 (16)

### 5‐year follow‐up

As shown in Table 2, 53% (*n* = 72) of the initial 135 reported less pain at 5 years from intra‐articular AD‐MSCs injection (VAS scores range, 0‐3). Within this group, 41 patients (57%) had also received arthroscopy as part of their treatment, whereas the remaining 31 (43%) patients had only received an intra‐articular injection of AD‐MSCs. Furthermore, by adding all the sub‐categories of patients at 5 years from the intervention who did not have unicondylar or total knee replacement (regardless of the reason behind not having had surgery), 50 patients out of the 122 (41%) who had not undergone surgical treatment were symptomatic. Amongst the symptomatic patients, 22 had received arthroscopy, whereas 28 exclusively received intra‐articular AD‐MSCs. Out of the 122 (91%) individuals from the initial cohort who were yet to receive surgical treatment for their knee OA at 5 years, 44% reported receiving further non‐surgical treatment following the intra‐articular AD‐MSCs, specifically intra‐articular hyaluronic acid, PRP and Ozone injections. The remaining 56% of patients had received no further treatment. Characteristics of the study population (122 patients) who had not yet received knee arthroplasty are shown in Table 2.

These 68 patients who had not received further treatment at 5 years from the procedure were asked to complete WOMAC questionnaires at 5 years from the procedure during routine follow‐up appointments. Demographics for patients who did not receive further non‐surgical treatment can be found in Table 3, compared to patients who did receive further non‐surgical management. Characteristics of the study population (Arthroscopy vs. No Arthroscopy) and WOMAC scores are shown in Table 4.

**Table 3:** Patient demographics subdivided between patients who received further non‐surgical treatment and patients who received no further treatment.

	Patients who received further non‐surgical treatment (*n* = 54)	Patients who did not receive further non‐surgical treatment (n = 68)	*p* **value**
Age (years)	62 (8)	63 (8)	0.38
Patient sex (female)	36 (67)	43 (63)	0.71
BMI (kg/m^2^)	27.5 (3.8)	28.6 (3.5)	0.13
Arthroscopy	23 (43)	40 (59)	0.10

*Note*: Values are presented as mean (standard deviation) or count (proportion). Differences between groups were calculated using analysis of variance and the two‐tailed Fisher's exact test, as appropriate.

Abbreviation: BMI, body mass index.

**Table 4:** Patient demographics and WOMAC scores for patients who did not receive further treatment during the 5‐year follow‐up period.

	Arthroscopy + MSC injection (*n* = 40)	MSC injection alone (*n* = 28)	*p* **value**
Age (years)	62 (8)	66 (8)	0.055
Patient sex (female)	24 (60)	19 (68)	0.61
Body mass index (kg/m^2^)	29.2 (3.3)	27.7 (3.7)	0.09
WOMAC × Pain	9 (3)	11 (3)	0.012
WOMAC × Stiffness	3 (1)	4 (2)	0.025
WOMAC × Function	34 (6)	43 (8)	<0.0001
WOMAC × Total score	46 (8)	58 (9)	<0.0001

*Note*: Values are presented as mean (standard deviation) or count (proportion). Differences between groups were calculated using analysis of variance and the two‐tailed Fisher's exact test, as appropriate.

Abbreviations: MSC, mesenchymal stromal cell; WOMAC, Western Ontario and McMaster University Osteoarthritis Index.

## DISCUSSION

The main findings of this study were that patients who received arthroscopy (intervention cohort) had more favourable results at 5 years, as they demonstrated superior WOMAC overall and functionality scores compared to the control cohort. However, non‐surgical re‐intervention rates were comparable between the two cohorts.

MSCs have the potential to regenerate the articular cartilage [7]. This was previously considered to be an impossible task due to the lack of vascularization of this type of tissue. However, recent in vivo studies have shown good cartilage formation and regeneration with AD‐MSCs [18]. While AD‐MSCs seem promising, previous studies have been disparate in terms of the usage of AD‐MSCs in the setting of knee OA. Specifically, a previous meta‐analysis suggested that AD‐MSCs should be considered as an additional treatment for knee OA rather than used independently, whereas a later systematic review showed that intra‐articular AD‐MSCs injection can reduce pain in patients suffering from knee OA in the first 6–12 months after the injection, even without adjuvant surgical intervention [22]. Contrary to this, a randomized controlled trial (RCT) with a 2‐year follow‐up was not able to find differences in terms of clinical and radiological outcomes in patients with knee OA following a single injection of intra‐articular autologous AD‐MSCs versus intra‐articular PRP [21]. Another RCT showed major improvements in pain, quality of life and cartilage regeneration at 12 months in patients who received intra‐articular AD‐MSCs compared to standard hyaluronic acid injections [12]. Notably, it is worth mentioning that neither of the two RCTs utilized therapeutic arthroscopies in addition to the injections. The combination of using therapeutic arthroscopy with the adipose tissue injection may be pivotal to acknowledge, since Nin et al. [14] showed that arthroscopy alone was associated with higher levels of joint stiffness following knee replacement surgery, if performed within 1 year after the arthroscopic procedure. Our results do not seem to show this in patients who also received intra‐articular AD‐MSCs, once again highlighting the probable positive long‐term effects of the procedure. Song et al. [16] found that AD‐MSCs treatment for knee OA significantly reduced WOMAC and VAS scores at the 6‐ and 12‐month follow‐ups, respectively, compared to control groups who had received Hyaluronic Acid injections. They suggested that AD‐MSCs are an effective and safe treatment option for early‐stage knee OA. In a similar study, all patients reported a significant decrease in VAS scores after autologous AD‐MSCs within the first year of treatment [1]. Finally, Vasso et al. [17] also showed how injection of intra‐articular microfragmented adipose tissue following arthroscopic debridement was effective in improving both clinical and functional scores at the 2‐year follow‐up.

A major factor to take into consideration when assessing the effect and durability of intra‐articular AD‐MSCs is the initial severity of knee OA. There seems to be a tendency to avoid intra‐articular AD‐MSCs in symptomatic radiographic Kellgren–Lawrence [10] Grade 4 knees. For this reason, all patients who were enroled in our study had either early, mild, or moderate scores, as described previously. The severity of the symptoms before the procedure was more critical when assessing the effects of the intra‐articular AD‐MSCs. It was noted how patients with lower initial VAS scores appeared to have superior symptomatic benefits despite having more severe Kellgren–Lawrence scores. This places further importance on the clinical examination aspect of the first orthopaedic encounter, as heavily symptomatic patients with significantly reduced quality‐of‐life scores could have reduced benefits from the procedure. In line with our findings, other authors state that particular attention should be placed on patient selection before conducting further studies to assess the actual clinical significance of this procedure [11]. Our results showed that out of all the patients who reported improvements in pain control at 5 years from the procedure, a slight majority (57%) had also received arthroscopic treatment. Speculatively, intra‐operative chondroabrasion, loose body removal, and articular washout can improve the effect of stand‐alone intra‐articular AD‐MSCs. Moreover, AD‐MSCs on their own have already been deemed comparable to PRP injections [21]. This hypothesis is strengthened by our study results as the cohort who had also received arthroscopy had significantly lower overall and functionality WOMAC scores, highlighting once again the efficacy of combining arthroscopy and AD‐MSCs. Comparable results were reported by Giorgini et al. [6], who conducted a single‐centre retrospective study on 49 patients with Kellgren–Lawrence Grades III–IV and showed how only 8% had received knee replacement surgery at 2 years from AD‐MSCs injections in combination with knee arthroscopy. Similar to previous suggestions, the authors further support the notion that only patients with lower‐grade radiographic OA should be considered for this treatment, as high‐grade chondral lesions impacted the outcome negatively. In our study, after excluding patients who underwent replacement surgery in the 5‐year follow‐up period, 59% of the non‐operated cohort were still asymptomatic in their index knee. Additionally, 53% of the patient cohort, who were asymptomatic at 5 years, had not received further treatment for their knee OA. This provides encouraging data for using AD‐MSCs, regardless of associating arthroscopy as part of the treatment. Nevertheless, the non‐surgical re‐intervention rate was comparable amongst the two cohorts.

According to previous literature, the usage of AD‐MSCs is deemed clinically valuable for knee OA, although the majority of studies are limited to 12‐ to 24‐month period follow‐ups. Nevertheless, our long‐term follow‐up demonstrates that intra‐articular AD‐MSCs are safe and can be considered as an effective treatment option for early‐stage knee OA. In support of this, 94% of our cohort reported some benefits from the procedure, and 53% of the group still had satisfactory symptomatic control in the treated knee at 5 years. Our results favour associating knee arthroscopy with the intra‐articular AD‐MSCs to achieve better symptomatic control and longer‐lasting functionality benefits. However, more robust data and larger sample sizes are required to fully validate these findings. Based on the current evidence, it is also clear that higher‐grade Kellgren–Lawrence knees should not be considered for this treatment option. This further highlights the importance of appropriate patient selection when considering this type of treatment.

### Strengths and limitations

Our study had several limitations. First, we could not randomize patients into two separate groups (arthroscopy vs non‐arthroscopy) as it was a retrospective study. Furthermore, the patients were treated by two separate surgeons in two different institutions, which, despite using identical protocols, adds an external variable to the procedural outcomes. Moreover, two different methods were used to process the harvested adipose tissue, increasing the risk of further variability. In fact, the Lipogems® system uses a microsphere system to filter the adipose tissue, whereas the Lipocell® system uses a semipermeable membrane to obtain a similar result. Lastly, we had to extrapolate most of our data from pre‐existing databases and patient encounters, which were not always standardized, allowing for a large error margin. We did, however, also have various strengths to our study, including the use of a score (WOMAC) which is specifically designed to assess OA, the improvement in VAS scores and the fact that we had an abundant follow‐up time of 5 years, with detailed information about every patient including intra‐operative arthroscopic findings.

## CONCLUSION

Patients undergoing intra‐articular AD‐MSCs can expect about a 10% possibility of undergoing total or unicondylar knee replacement surgery within 5 years from the procedure (although a slightly higher percentage may have an indication for replacement), regardless of whether they undergo associated knee arthroscopy or not. Overall outcomes are superior in those patients who also received the arthroscopic debridement and lavage, as both WOMAC overall and functionality scores were superior compared to the non‐arthroscopy cohort at 5 years. Non‐surgical treatment re‐intervention rates were similar amongst the two cohorts. Patients with grade IV Kellgren‐Lawrence knees should not be considered for this treatment option.

## AUTHOR CONTRIBUTIONS

**Simone Giusti**: Conceptualization; data curation; writing—original draft; writing—review and editing; project administration. **Ezio Adriani**: Conceptualization; supervision. **Kristian Samuelsson**: Project administration; supervision. **Alice Laudisio**: Methodology, data curation **Alexandra Horvath**: Writing—review and editing. **Biagio Zampogna**: Conceptualization; writing—original draft; writing—review and editing; project administration. **Rocco Papalia**: Conceptualization; supervision.

## CONFLICT OF INTEREST STATEMENT

Kristian Samuelsson is a member of the Board of Directors of Getinge AB (publ) and medtech advisor to Carl Bennet AB. The other authors declare no conflicts of interest.

## ETHICS STATEMENT

All participants signed informed consent to be included in the observational study and for anonymous data regarding their procedures to be published for scientific purposes. Approval was gained from the institution's IRB (IRB 21.19 TS).

## Data Availability

All data can be made available upon reasonable request.
